# Effectiveness of lifestyle interventions for glycaemic control among adults with type 2 diabetes in West Africa: a systematic review and meta-analysis protocol

**DOI:** 10.1186/s13643-024-02555-8

**Published:** 2024-09-03

**Authors:** Ellen Barnie Peprah, Yasmin Jahan, Anthony Danso-Appiah, Abdul-Basit Abdul-Samed, Tolib Mirzoev, Edward Antwi, Dina Balabanova, Irene Agyepong

**Affiliations:** 1 Ghana College of Physicians and Surgeons, Accra, Ghana; 2 London School of Hygiene and Tropical Medicine, London, UK; 3 University of Ghana, Accra, Ghana; 4 Ghana Health Service, Accra, Ghana

**Keywords:** Type 2 diabetes, Physical activity, Diet modification, Nutrition, Exercise, West Africa, Glycaemic control

## Abstract

**Background:**

Lifestyle interventions are key to the control of diabetes and the prevention of complications, especially when used with pharmacological interventions. This protocol aims to review the effectiveness of lifestyle interventions in relation to nutrition and physical activity within the West African region. This systematic review and meta-analysis seeks to understand which interventions for lifestyle modification are implemented for the control of diabetes in West Africa at the individual and community level, what evidence is available on their effectiveness in improving glycaemic control and why these interventions were effective.

**Methods:**

We will review randomised control trials and quasi-experimental designs on interventions relating to physical activity and nutrition in West Africa. Language will be restricted to English and French as these are the most widely spoken languages in the region. No other filters will be applied. Searching will involve four electronic databases — PubMed, Scopus, Africa Journals Online and Cairn.info using natural-language phrases plus reference/citation checking.

Two reviewers will independently screen results according to titles and abstracts against the inclusion and exclusion criteria to identify eligible studies. Upon full-text review, all selected studies will be assessed using Cochrane’s Collaboration tool for assessing the risk of bias of a study and the ROBINS-I tool before data extraction. Evidence will be synthesised narratively and statistically where appropriate. We will conduct a meta-analysis when the interventions and contexts are similar enough for pooling and compare the treatment effects of the interventions in rural to urban settings and short term to long term wherever possible.

**Discussion:**

We anticipate finding a number of studies missed by previous reviews and providing evidence of the effectiveness of different nutrition and physical activity interventions within the context of West Africa. This knowledge will support practitioners and policymakers in the design of interventions that are fit for context and purpose within the West African region.

**Systematic review registration:**

This systematic review has been registered in the International Prospective Register for Systematic Reviews — PROSPERO, with registration number CRD42023435116. All amendments to this protocol during the process of the review will be explained accordingly.

**Supplementary Information:**

The online version contains supplementary material available at 10.1186/s13643-024-02555-8.

## Background

Diabetes is a chronic disease estimated to affect 537 million adults worldwide. According to the World Health Organization, 1.5 million deaths are directly attributable to diabetes annually, and this disproportionately affects populations in developing countries. This same population is often at higher risk of late diagnosis, poor clinical management and its associated microvascular and/or cardiovascular complications [1].

The West African region — home to 16 developing economies — is still reeling from the impact of the COVID-19 pandemic. The rapidly changing sociocultural environment, demographics and economic conditions further threaten to worsen the burden of noncommunicable diseases such as diabetes within the region [2, 3]. With less than 7 years to meet the United Nations Sustainable Development Goal 3.4 target of reducing by a third premature mortality from noncommunicable diseases, greater investment in interventions that bridge the gaps in service delivery, programme design and policy implementation will be required.

The 2023 Standards of Care in Diabetes of the American Diabetes Association (ADA) names, among many others, physical activity and medical nutrition therapy as interventions to facilitate positive health behaviours to improve outcomes for diabetes [4]. Physical activity includes all movements that increase energy expenditure such as walking, housework, gardening, swimming, dancing, yoga, aerobic activities and resistance training. Exercise, on the other hand, is structured and tailored towards improving physical fitness. Interventions for physical activity and exercise are both recommended for better glycaemic control [5]. ADA recommends at least 150 min or more of moderate to vigorous exercise a week and encourages an increase in non-sedentary physical activity among people living with type 2 diabetes. The goal of interventions for nutrition therapy is to manage weight, achieve individual glycaemic control targets and prevent complications. ADA recommends that nutrition therapy and counselling, under the guidance of a registered dietician, is administered to patients with type 2 diabetes with emphasis on managing energy balance, dietary protein, carbohydrate and fat intake and alcohol consumption [4].

### Rationale

There is evidence available supporting the effectiveness of physical activity and nutrition interventions to achieve glycaemic control and improve overall cardiometabolic health in other populations [6–8]. However, there is not much evidence of its effectiveness in the West African population. Those that are documented in literature exist in fragmented, regional spaces, and the West African context could be easily lost in larger studies such as Sagastume et al. [9]. O’Donoghue and colleagues [10] reviewed randomised control trials on lifestyle interventions from low- and middle-income countries. However, the sheer geographical breadth of studies represented within the review, the diversity of populations, differences in health system structures and priorities [11] and cultural and socio-economic contexts included in the review pose a challenge to generalisation of study findings to the West African population. Also, controversies remain on what type of nutrition therapy or meal plans work best for people with diabetes [12], whether structured self-management education yields greater benefit for patients [13] and whether exercise, its duration or intensity has varying effect on glycaemic control in patients with diabetes [14, 15]. The aforementioned present the need to assemble existing studies and synthesise what is known about their effectiveness. Knowledge of what exists would shape future interventions for diabetes control in West Africa.

### Objectives

This review will seek to address the following questions:

1. Which individual-level interventions for lifestyle modification are available for the control of type 2 diabetes in adults West Africa?
2. What is the effectiveness of the available individual level interventions for lifestyle modification in glycaemic control?
3. Which community-level interventions for lifestyle modification are implemented for the control of diabetes in West Africa?
4. What is the effectiveness of community level interventions for lifestyle modification in improving glycaemic control?
5. Which factors influence the effectiveness of glycaemic control interventions at the individual and community level?

## Methods

### Criteria for considering studies for this review

The Population, Intervention, Comparison, Outcome and Studies (PICOS) framework will be used in determining inclusion for the study.

#### Population

Adults aged 18 years and older living in West Africa with previously or newly diagnosed type 2 diabetes. We will not consider type 1 diabetes and paediatric and gestational diabetes mellitus.

#### Intervention

All lifestyle interventions relating to physical activity and nutrition will be considered. Physical activity will include low, moderate and high intensity exercises. Non-sedentary everyday movement such as walking, gardening and housework will be considered so long as it is delivered in a regimen and has been measured. Interventions for nutrition will include vegetarian, low carbohydrate diet, low fat or plant-based diet. For the purpose of this review, interventions for alcohol reduction will be considered as a part of nutrition. The duration of intervention could be short-term interventions which we define as 3 months or less or long-term intervention which we define as greater than 3 months. We define individual-level interventions as those targeted at the individual patient, such as one-on-one counselling or structured education programmes delivered to an individual. Community-level interventions are those implemented at the broader community or population level, such as public awareness campaigns and community-based physical activity programmes. In all situations, interventions could be provider-led, and group-based or individually based activities will be considered in the review.

#### Control

The control will be usual care or no intervention.

#### Outcome

The primary outcome of interest to this review is glycaemic control as indicated by glycated haemoglobin (HBA1c) values. Despite objections to the preference of HBA1c for diagnosing diabetes by some researchers based on the cost and biological variation [16], it is generally regarded as a reliable metric for glycaemic improvement in clinical trials [17]. We will say an intervention improves glycaemic control when there is a clinically significant reduction of HbA1c of greater 5 mmol/mol or 0.5% of HbA1c from pre-intervention baseline [18]. If there is a non-clinically significant reduction in HbA1c of less than 5 mmol/mol or 0.5% of HbA1c, no reduction or an increase in HbA1c from pre-intervention baseline, we will say that intervention does not improve glucose control.

#### Studies

Eligible study designs will be limited to randomised control trials and quasi-experimental studies such as pretest and posttest study designs, nonequivalent control group designs and controlled observational studies that attempt to establish causal relationships between the intervention and the outcomes.

### Search

We will search four online databases (PubMed, Scopus, Africa Journals Online and Cairn.info) for articles published from 2000 to 31st August 2024 We will also search websites of relevant government agencies and non-governmental organisations such as PATH and Sante Diabete for programme reports, evaluations and relevant publications and clinical trial registries for ongoing or recently completed trials (summarised in Additional File 1, PRISMA_2020_Search flowchart.docx attached). In order not to miss any relevant study, we will also search through the reference list and bibliographies of included studies.

### Search strategy

Search terms we will use include “diabetes”, “lifestyle modification”, “physical activity”, “nutrition” and their synonyms, and MESH terms. (Additional File 2, Search strategy.docx) detail the full search strategy and a sample search for PubMed. Language will be restricted to English and French as these are the most widely used for scholarly publications and reports within the region. No other filters will be applied. A search alert will be created to update on any new studies, while the search and screening process is ongoing.

### Study selection and management

Two reviewers will independently screen search results according to titles and abstracts against the inclusion and exclusion criteria to identify eligible studies (see Additional file 3, Algorithm for Screening.docx). Duplicates and irrelevant titles and abstracts will be removed. A third reviewer will settle discrepancies through a consensus. A full-text review of all selected studies will then be conducted against the inclusion criteria to identify studies to be included for analysis. Search results will be managed using the Rayyan software platform to facilitate the screening process.

### Study risk-of-bias assessment

All selected studies will be assessed using Cochrane’s Collaboration tool for assessing the risk of bias of a study. For the risk-of-bias assessment of non-randomised studies, we will use the ROBINS-I tool. Judging from quotes from the authors, two independent reviewers will rate studies as either low risk, high risk or unclear, and a third reviewer will settle discrepancies if there are any. A sensitivity analysis will be conducted to evaluate the impact of high-risk studies on the overall analysis before a decision to exclude studies will be done.

### Data extraction and management

For each of the studies selected, the following data will be extracted independently on a data collection form in Microsoft Excel by two reviewers: (1) first author’s last name; (2) year of publication; (3) country; (4) study setting; (5) characteristics of participants, sample size and mean age; (6) type of intervention, frequency and duration; (7) characteristics of control group; (8) pre-intervention baseline HbA1c; (9) post-intervention HbA1c; (10) any other outcomes if reported; and (11) author’s conclusions. We will contact authors for missing data or to clarify data. We will first attempt to contact the corresponding authors of the included studies via email, providing a clear timeline for their response. If the authors do not respond within 4 weeks, we will send a follow-up email. If the authors do not respond, we will proceed with the data synthesis and clearly report the missing information and its potential impact on the overall findings in the limitations section of the review.

### Strategy for data synthesis

We will estimate the effect of the intervention using the relative risk for the number achieving glycaemic control as our primary outcome. If other effect estimates are provided, we will convert between estimates where possible. Measures of precision will be at 95% confidence intervals which will be computed using the participants per treatment group rather than the number of intervention attempts. Study authors will be contacted if there is the need for further information or clarification about methods used in analysing results. If the author of selected articles cannot be reached for clarification, we will not report confidence intervals or *p*-values for which clarification is needed. When both pre-intervention baseline and endpoint measures are reported, endpoint measures and their standardised deviation will be used.

We will conduct a meta-analysis when the interventions and contexts are similar enough for pooling. Since heterogeneity is expected a priori due to age, sex and study setting, i.e. whether urban or rural, we will estimate the pooled treatment effect estimates and its 95% confidence interval controlling for these variables. Forest plots will be used to visualise the data and extent of heterogeneity among studies. We will conduct a sensitivity analysis to explore the influence of various factors on the effect size of only the primary outcome, that is glycaemic control. Any post hoc sensitivity analyses that may arise during the review process will be explained in the final report.

We will use a cluster-based analysis when analysing interventions at the community level. When both individual- and cluster-level factors are reported, we will use cluster-level data for our analysis taking into consideration their design effect. We intend to perform a thematic, qualitative analysis in determining the factors that influence the effectiveness of identified interventions at the community level.

## Discussion

We anticipate retrieving data about the West African context on the effectiveness of physical activity and nutrition interventions on improving glycaemic control in patients living with an established type 2 diabetes. This information will guide practitioners and policymakers to design interventions that are fit for context and purpose within West Africa and Africa, by extension.

### Supplementary Information

Additional file 1: Figure 1. PRISMA 2020 flow diagram for new systematic reviews which included searches of databases, registers and other sources.

Additional file 2. Describes search concepts, includes a sample search for PubMed. Table 1. PubMed search strategy.

Additional file 3: Figure 2. Decision-making flowchart for screening.

## Data Availability

The datasets used and/or analysed during the current study are available from the corresponding author on reasonable request.

## Abbreviations

ADA: American Diabetes Association
PRISMA: Preferred Reporting Items for Systematic Reviews and Meta-Analyses
PICOS: Population, Intervention, Comparison, Outcome and Studies
HBA1c: Glycated haemoglobin
