# Supplementary material for: Effectiveness of lifestyle interventions for glycaemic control among adults with type 2 diabetes in West Africa: a systematic review and meta-analysis protocol
Source: Syst Rev. 2024 Sep 3;13:226. doi: 10.1186/s13643-024-02555-8 (PMC11370026; doi:10.1186/s13643-024-02555-8)
Supplement: Supplementary file 1 — Additional file 1: Figure 1. PRISMA 2020 flow diagram for new systematic reviews which included searches of databases, registers and other sources. [file 13643_2024_2555_MOESM1_ESM.docx]

**Identification of studies via other methods**

**Identification of studies via databases and registers**

Records identified from:

Websites (n = )

Organisations (n = )

Citation searching (n = )

etc.

Records removed *before screening*:

Duplicate records removed (n = )

Records marked as ineligible by automation tools (n = )

Records removed for other reasons (n = )

Records identified from:

PubMed (n = )

Scopus (n = )

Africa Journals Online (n = )

Cairn.Info (n=)

**Identification**

Records excluded(n = )

Not Type 2 DM diagnosed (n = )

Not West Africa (n = )

Not Adult (n=)

Not Intervention (n = )

Not Physical Activity-related (n=)

Not Nutrition- related (n=)

Records screened

(n = )

Reports not retrieved

(n = )

Reports sought for retrieval

(n = )

Reports sought for retrieval

(n = )

Reports not retrieved

(n = )

**Screening**

Reports assessed for eligibility

(n = )

Reports excluded:

Reason 1 (n = )

Reason 2 (n = )

Reason 3 (n = )

etc.

Reports assessed for eligibility

(n = )

Reports excluded: (n= )

Reason:

Study Design (n=)

No HbA1c results(n= )

Other:

Studies included in review

(n = )

Reports of included studies

(n = )

**Included**

*From:*  Page MJ, McKenzie JE, Bossuyt PM, Boutron I, Hoffmann TC, Mulrow CD, et al. The PRISMA 2020 statement: an updated guideline for reporting systematic reviews. BMJ 2021;372:n71. doi: 10.1136/bmj.n71. For more information, visit: <http://www.prisma-statement.org/>
