# Supplementary material for: Effectiveness of lifestyle interventions for glycaemic control among adults with type 2 diabetes in West Africa: a systematic review and meta-analysis protocol
Source: Syst Rev. 2024 Sep 3;13:226. doi: 10.1186/s13643-024-02555-8 (PMC11370026; doi:10.1186/s13643-024-02555-8)
Supplement: Supplementary file 3 — Additional file 3: Figure 2. Decision-making flowchart for screening. [file 13643_2024_2555_MOESM3_ESM.docx]

Figure 2: Decision-making flowchart for screening

**STEP 2: Does the study focus on**  lifestyle interventions relating to physical activity and nutrition **in this age group**

**STEP 3: Is this a randomized control trial or a quasi-experimental study or review**

Label: Wrong Study type

Label: Wrong topic. Not related to physical activity or nutrition

**STEP 3: Was the study conducted in a country in West Africa (WA)**

Label: Wrong setting /context. Not WA

**STEP 1: Does the study focus on** Adults (Age 18 and above) living with diabetes

Label: Wrong demographic group.

No. Exclude

Yes, include

No. Exclude No. Exclude

Yes, include

No. Exclude

E.g.Others:

commentaries, opinions

Yes, include

No . exclude

No. exclu

Yes, include

Analysis
